# Synergy between histone deacetylase inhibitors and DNA-damaging agents is mediated by histone deacetylase 2 in colorectal cancer

**DOI:** 10.18632/oncotarget.9887

**Published:** 2016-06-07

**Authors:** Samer Alzoubi, Leigh Brody, Sunniyat Rahman, Anne-Laure Mahul-Mellier, Nicolas Mercado, Kazuhiro Ito, Mona El-Bahrawy, Andrew Silver, Alan Boobis, Jimmy D. Bell, Nabil Hajji

**Affiliations:** ^1^ Department of Medicine, Division of Experimental Medicine, Centre for Pharmacology & Therapeutics, Toxicology Unit, Imperial College London, London, UK; ^2^ Department of Life Sciences, Research Centre for Optimal Health, University of Westminster, London, UK; ^3^ Laboratory of Molecular and Chemical Biology of Neurodegeneration, Brain Mind Institute, Ecole Polytechnique Fédérale de Lausanne, Lausanne, Switzerland; ^4^ Airway Disease Section, National Heart and Lung Institute, Imperial College, London, UK; ^5^ Department of Histopathology, Imperial College London, London, UK; ^6^ Colorectal Cancer Genetics, Centre for Genomics & Child Health, Blizard Institute, Barts and The London School of Medicine and Dentistry, London, UK

**Keywords:** colorectal cancer, histone acetylation, drug resistance and in vivo imaging, HDAC2, p53

## Abstract

Previous studies have associated the overexpression of histone deacetylase 2 (HDAC2) and the presence of *TP53* mutations with the progression to advanced stage drug resistant colorectal cancer (CRC). However, the mechanistic link between HDAC2 expression and the *TP53* mutational status has remained unexplored. Here, we investigated the function of HDAC2 in drug resistance by assessing the synergistic effects of DNA-targeted chemotherapeutic agents and HDAC inhibitors (HDACis) on two *TP53*-mutated colorectal adenocarcinoma CRC cell lines (SW480 and HT-29) and on the *TP53*-wild type carcinoma cell line (HCT116 p53+/+) and its *TP53* deficient sub-line (HCT116 p53−/−). We showed that in the untreated SW480 and HT-29 cells the steady-state level of HDAC2 was low compared to a *TP53*-wild type carcinoma cell line (HCT116 p53+/+). Increased expression of HDAC2 correlated with drug resistance, and depletion by shRNA sensitised the multi-drug resistance cell line HT-29 to CRC chemotherapeutic drugs such as 5-fluorouracil (5-FU) and oxaliplatin (Oxa). Combined treatment with the HDACi suberoylanilide hydroxamic acid plus 5-FU or Oxa reduced the level of HDAC2 expression, modified chromatin structure and induced mitotic cell death in HT-29 cells. Non-invasive bioluminescence imaging revealed significant reductions in xenograft tumour growth with HDAC2 expression level reduced to <50% in treated animals. Elevated levels of histone acetylation on residues H3K9, H4K12 and H4K16 were also found to be associated with resistance to VPA/Dox or SAHA/Dox treatment. Our results suggest that HDAC2 expression rather than the p53 mutation status influences the outcome of combined treatment with a HDACi and DNA-damaging agents in CRC.

## INTRODUCTION

Altered expression of histone deacetylases (HDAC) is associated with cancer initiation, progression, metastasis and drug resistance [1]. This has led to clinical trials involving HDACs inhibitors (HDACis) as single or combined treatments with DNA damaging agents [2]. Moreover, over-expression of HDACs has been implicated in the silencing of tumour suppressor genes [3], the promotion of genotoxic protection and resistance to DNA damaging agents in many cancer types, possibly through the activation of non-histone proteins required for DNA stability and higher-order chromatin organisation [4].

Colorectal cancer (CRC) is a common gastrointestinal malignancy and the second most common cause of cancer-related deaths in the Western world [5]. Patients with metastatic CRC (mCRC) have poor prognosis [6] and both adjuvant and standard cancer therapies remain sub-optimal in a significant number of cases due to treatment resistance [7]. Genetic and epigenetic alterations are evident in CRC initiation, progression and in resistance to conventional drugs [7, 8]. The tumour suppressor gene *TP53* is often inactivated by loss of function mutations in CRC [9], although other genetic (*APC* and *RAS*) or epigenetic alterations (e.g. DNA methylation promoter silencing) may compromise *TP53* response to DNA damage [10].

Aberrant expression patterns of HDAC2 are found in a number of cancers including CRC [11]. Over-expression of HDAC2 occurs early at the premalignant polyp stage of CRC [12] and correlates with a poor prognosis in advanced stage disease [13]. The presence of HDAC2 frame shift mutation in cancers from individuals with hereditary non-polyposis colorectal cancer syndrome caused a loss of HDAC2 protein expression and enzymatic activity and rendered tumour cells more resistant to trichostatin A, a pan-HDACi [14].

The relationship between the mutational status of P53 and HDAC2 overexpression is not well understood in CRC drug response and the underlying molecular mechanisms of HDACis remain poorly explored [15]. HDACis are effective therapeutic anticancer agents via multiple mechanisms, which make them very attractive agents not only for monotherapy but also for combination therapy with other anticancer modalities. HDACis can modulate cellular responses to DNA damaging agents including ionising and ultraviolet radiation, and chemotherapeutic drugs [16]. Many HDACi / DNA damaging agent combination strategies are both effective and synergistic whereas others are ineffective or antagonistic with unclear mechanistic reasons for these effects [17]. Hence, understanding the mechanisms of HDACi resistance is crucial to develop more effective combination strategies for the future [18].

The aim of our study was to investigate the role of HDAC2 in drug resistance and to assess its impact on CRC cell lines with varied *TP53* mutation states, (wild-type, null and mutated) in response to the combined treatment with DNA-targeted chemotherapeutics agents and HDACis. Our results suggest that HDAC2 expression rather than the p53 mutation status influences the outcome of combined treatment with a HDAC inhibitor and DNA-damaging agents in CRC. Furthermore, elevated levels of histone acetylation were found to be associated with drug resistance in our cellular models. This is particularly significant as we show that HDAC2 expression is increased in moderately differentiated human metastatic colorectal carcinomas in the liver compared with normal tissues. Taken together, our results demonstrate the potential of using HDAC2 expression levels as a biomarker in understanding the effectiveness of combined treatment.

## RESULTS

### The response of wild type, null, and mutated *TP53* CRC cell lines to DNA damaging agents

**Figure 1:**
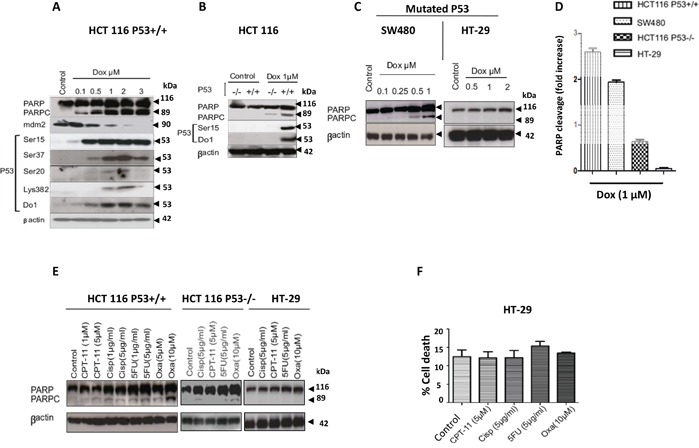
Characterization of WT, null, and mutated P53 CRC cell lines response to DNA damaging agents **A.** HCT116 P53+/+ cells were treated with dose-increase of Dox for 24hr and checked for PARP cleavage, also activation and stabilization of P53 were analyzed by western blotting. **B.** Comparison of PARP cleavage upon Dox 1μM between HCT116 P53+/+ and HCT116 P53−/−. **C.** Assessment of PARP cleavage in SW480 and HT-29cells lines upon a dose-increase of Dox. **D.** HCT116 P53+/+, HCT116 P53−/−, and HT-29 cells were treated with 1μM Dox and proteins cell lysate were separated using SDS-PAGE and detected by WB analysis. Quantification of PARP cleavage detected by western blotting was performed by ImageJ software and based on the ratios of normalized cleaved PARP by βactin and normalized uncleaved PARP. (Fold changes are the average of three independent of three independent experiments (*n*=3)). **E.** HCT116 P53+/+ cells, HCT116 P53−/− and HT-29 cells were treated with chemotherapeutic drugs for 24hr. Cells were lysed and the proteins separated using SDS-PAGE and detected by WB analysis. The cleavage of PARP was assessed using the appropriate antibodies. **F.** HT-29 cell death investigated by propidium iodide (PI) staining and flow cytometry analysis (mean ± S.E.M. of three independent experiments (*n*=3)), the cell death following treatments with (5-FU and Oxa) were not significant in comparison with the control (P=0.55), and this supports the results western blot for these treatments (no PARPc). In all the western blotting experiments βactin was used as a loading control.

Mutations in *TP53* tumour suppressor gene are well-known events, which take place in the most aggressive cancers. However, the significance of mutated *TP53* in drug resistance is controversial in many cancers. In this study, we investigated the role of p53 in the induction of CRC cell death by DNA damaging agents in the presence or absence of wild-type p53. The wild type (WT) *TP53* cell line HCT116 (HCT116 p53+/+) was treated with increasing concentrations (0.1-3 μM) of the DNA damaging agent doxorubicin (Dox), a topoisomerase II inhibitor. Incubation of HCT116 p53+/+ cells with 0.5μM Dox was sufficient to phosphorylate multiple p53 serine residues (Ser15, Ser37, and Ser20). These post-translational modifications (PTM) led to p53 accumulation in cells (Figure 1A). Dox was able to induce apoptosis in concentration-dependent manner as shown by PARP cleavage (PARPc) (Figure 1A). Acetylation of p53 at residue K382 as contributor of its activation was observed after exposure to 1-3μM Dox followed by substantial increase of PARPc (Figure 1A). Therefore, we sought to determine the role of p53 in controlling the sensitivity to Dox. The *TP53* WT (HCT116 p53+/+) and null isogenic *TP53* (HCT116 p53−/−) cell lines were treated with 1μM Dox and assessed for PARPc by immunoblotting (Figure 1B). HCT116 p53−/− cells were less sensitive to 1μM Dox treatment and showed less cell death in comparison with HCT116 p53+/+ suggesting that in absence of p53, the cells were less sensitive to Dox treatment compared to HCT116 p53+/+ cells (Figure 1A and 1B). To confirm the importance of the *TP53* gene in regulating DNA damage responses, SW480 and HT29 cells with *TP53* mutations were used. SW480 has two mutations in *TP53*: one is similar to that in HT-29 (mutation in TP53 DNA-binding site, codon 273; and the second is located in the P53 oligomerisation domain, codon 309). The Dox sensitivity of the two mutated cell lines were compared to HCT116 p53+/+ and HCT116 p53−/− cell lines. HT-29 cells were treated with 0.5 μM, 1μM and 2 μM Dox which all failed to induce PARPc (Figure 1C). Surprisingly, PARPc was observed in SW480 treated with much lower 0.5 μM and 1μM concentrations of Dox (Figure 1C and 1D). To determine if the difference in sensitivity was Dox specific, additional drugs were tested including cisplatin and camptothecin, and also 5-fluorouracil (5-FU) and Oxaliplatin (Oxa) both of which are first-line chemotherapeutic agents used to treat patients with Dukes' B and C CRC. Low and high concentrations were selected for each drug treatment. As HT-29 exhibited more resistance to Dox compared to the other cell lines, only the high concentrations of selected drugs were used. HT-29 cells exhibited extreme resistance to all drugs tested compared to the other cell lines, HCT116 p53+/+ and HCT116 p53−/−, as demonstrated by PARPc (hallmark of apoptosis) (Figure 1E and 1F).

### HDAC inhibitors in combination with DNA damaging agents induce distinct levels of sensitivity to the induction of cell death in CRC cells

**Figure 2:**
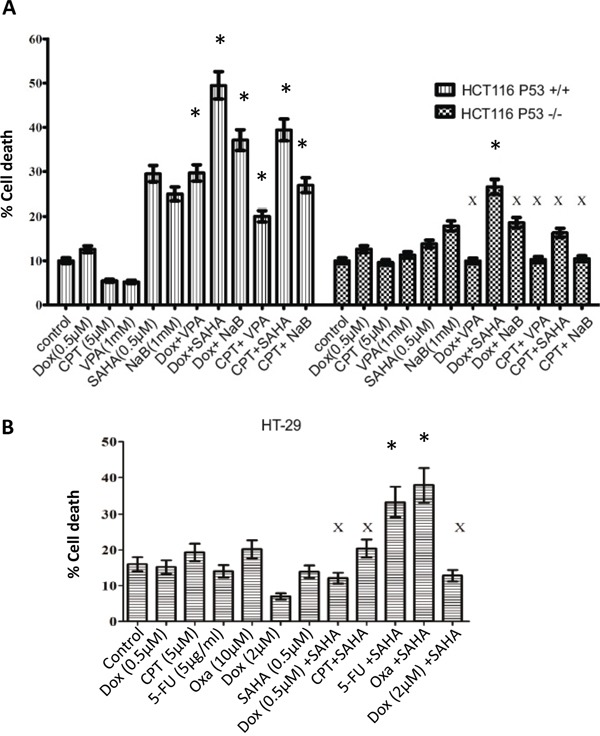
Combined treatment with distinct HDAC inhibitors and DNA damaging agents induces different levels of sensitivity in CRC cells **A.** HCT116 p53+/+, HCT116 p53−/− cells were treated with a single treatment of Dox (0.5μM) and Camptothecin-11 (CPT-11) (5μM) or combined treatment with VPA (1 mM), SAHA (0.5μM), and sodium butyrate (NaB) (1 mM) for 24 h and then cells were prepared for the cell death analysis by flow cytometry using Propidium iodide (PI) staining. We calculated the combination index (CI) according to the Chou-Talalay method using Calculsyn software (Biosoft, Cambridge, UK). Chou-Talalay method for drug combination was based on the median-effect to defined synergy and antagonism [38]. This method offered synergistic (CI < 1), and antagonistic effect (CI > 1) in drug combinations. Error bars represent ± S.E.M of three independent experiments (*n*=3). **B.** HT-29 cell death analysis by flow cytometry after 24 hours treatment with (Dox, CPT, 5FU), SAHA or their combination. Cells were harvested and stained with Propidium iodide (PI) to determine cell death using FACS analysis. * denotes a synergistic effect and X denotes an antagonistic effect. Error bars represent ± S.E.M of three independent experiments (*n*=3).

To characterise whether HDACis work synergistically in combination with DNA damaging agents; three HDAC inhibitors were used, suberoylanilide hydroxamic acid (SAHA or vorinostat), as an example of a pan HDACi active against class I and class II HDACs; the short-chain fatty acid sodium butyrate (NaB) and valproic acid (VPA), a class I and class IIa HDAC inhibitor. To determine whether any synergistic effects, low concentrations of DNA damaging agents were selected and combined with the HDACis. HCT116 p53+/+ and HCT116 p53−/− were treated with a single treatment of Dox (0.5μM) and Camptothecin-11 (CPT-11) (5μM) or combined with VPA (1 mM), SAHA (0.5μM), and NaB (1 mM) for 24 h and then analysed for cell death by flow cytometry. Significant cell death was observed following SAHA and NaB as a single treatment in HCT116 p53+/+ but only a slight increase in HCT116 p53−/− (Figure 2A). The combined treatments in HCT116 p53+/+ induced a synergistic effect on cell death in comparison with the single treatment, whereas HCT116 p53−/− showed a synergistic effect only with Dox/SAHA (Figure 2A). However, in HCT116 p53−/− cells an antagonistic effect on cell death was observed with the Dox/VPA and CPT/NaB combinations. Antagonistic effects on HT-29 cell death were observed with Dox at 0.5 μM and 2 μM combined with SAHA and CPT/SAHA (Figure 2B). Interestingly, only the combined treatments, SAHA/5-FU and SAHA/Oxa showed synergistic effects on HT-29 cell death (Figure 2B).

### HDAC2 expression after drug treatment with DNA damaging agents singly or combined with HDACis

**Figure 3:**
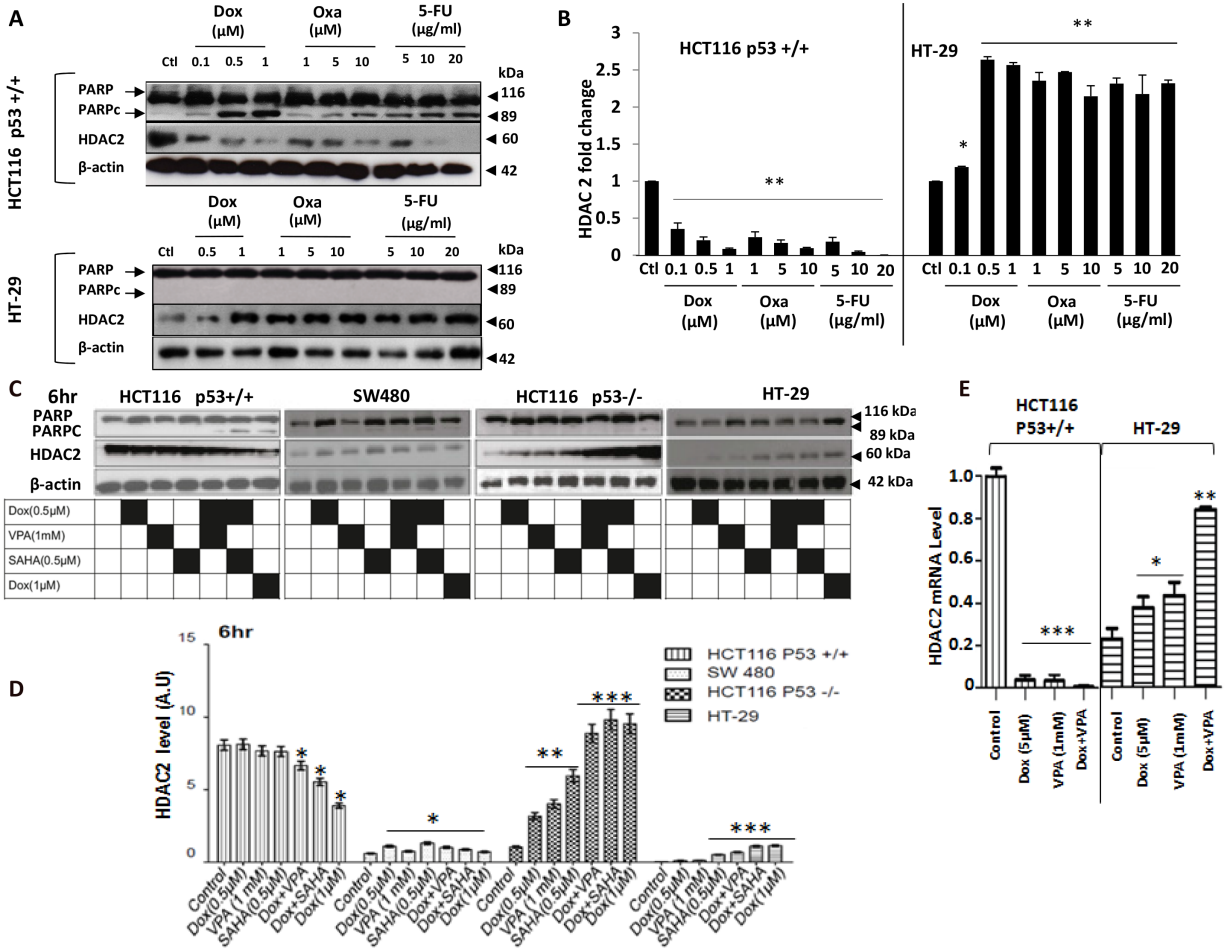
Early effects of Dox combined to SAHA or VPA on CRC cell lines **A** and **B.** HCT116 and HT-29 cells were treated for 24 hr treatment with dose-increase of Dox, Oxa or 5-FU. Cells were lysed and the proteins separated using SDS-PAGE and detected by WB analysis. The cleavage of PARP (PARPc) and the protein level of HDAC2 were assessed and quantified using the appropriate antibodies. Actin was used as a loading control. **C.** The four cell lines (HCT116 p53+/+, HCT116 p53−/−, SW480, and HT-29) were treated for 6 hours with Dox, VPA or SAHA only or as a combined treatment. Cells were lysed and the proteins separated using SDS-PAGE and detected by WB analysis. The PARPc and the protein level of HDAC2 were assessed using the appropriate antibodies. Actin was used as a loading control. **D.** Four cell lines were treated with Dox either singly or combined with SAHA or VPA for 6 hours and cells were lysed and the proteins separated using SDS-PAGE and detected by WB analysis. HDAC2 protein levels were assessed using the appropriate antibodies and quantified using ImageJ software. **E.** Total RNA was extracted from HCT116 p53+/+ and HT-29 cells and *HDAC2* mRNA expression level was measured by quantitative *RT-PCR* using the primer: forward primer (5′-3′) GT GAG ATT CCC AAT GAG TTG C. reverse primer (5′-3′) GGT AAC ATG CGC AAA TTT TCA A. Error bars represent ± S.E.M.; n=3 independent experiments. Test, t-test, * for *p<.005*, ** for *p*<.001, and *** for *p*<.0001.

HDAC2 overexpression and p53 mutations are significantly associated with advanced stages and poor prognosis in CRC patients [13]. However, the relationship between HDAC2 and p53 status is not completely understood nor the role and regulation of HDAC2 expression level upon drug treatments. Therefore, we investigated the relationship between HDAC2 expression level and the effect of DNA damaging agents as single treatments or combined with HDACis in CRC cells lines. Cells were exposed for 24 h to increasing concentrations of Dox, Oxa and 5-FU followed by the assessment of HDAC2 and PARPc protein level by immunoblotting. We observed a dose-dependent decrease in HDAC2 protein expression (P<0.001), which was inversely proportional to PARPc increase in HCT116 P53+/+ cells (Figure 3A and 3B). In contrast, upon exposure to different concentrations of the drugs, HT-29 cells exhibited extreme resistance to PARPc induction and a significant increase in HDAC2 protein expression (P<0.001) (Figure 3A and 3B).

The effect of combined treatment on HDAC2 protein levels was investigated in the four CRC cell lines with different *TP53* mutational status: HCT116 p53+/+, HCT116 p53 −/−, SW480, and HT-29. All cell lines were treated for 6 and 24 hours with the different combinations of the drugs. At 6 hours, the p53+/+ cell line exhibited sensitivity to the VPA/Dox and SAHA/Dox combinations, but not to the single treatment as measured by PARPc (Figure 3C). In HCT116 p53+/+ cell death correlated with a significant decrease in HDAC2 expression (P<0.001) (Figure 3C and 3D). However, null p53, SW480 and HT-29 showed a marked increase of HDAC2 following single or combined treatments, which correlated with resistance to the treatment (Figure 3C and 3D).

*HDAC2* mRNA levels were also affected by the drugs treatments. In HCT 116 p53+/+ cells, *HDAC2* mRNA was reduced significantly (P < 0.001) following Dox or VPA as single treatment or combined. In contrast HT-29 cells exhibited a significantly increased *HDAC2* mRNA expression (P < 0.001) upon the combined treatment Dox/VPA (Figure 3E).

**Figure 4:**
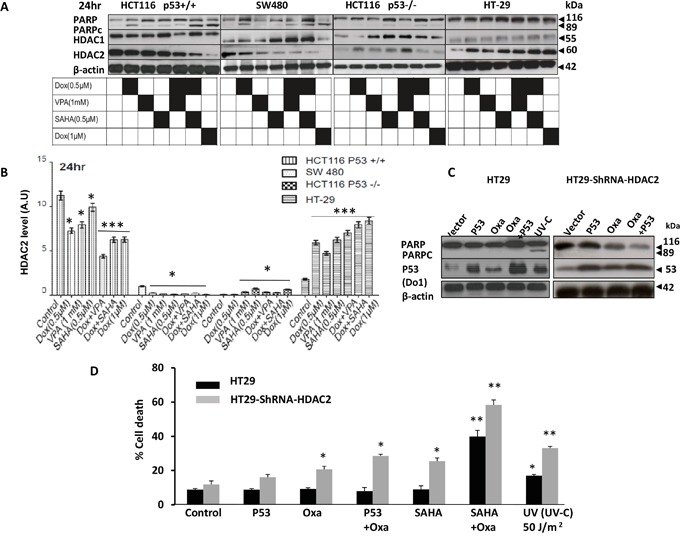
Characterisation of HDAC2 expression levels in distinct CRC cell lines and its relationship with P53 and resistance to HDACis combined with DNA damaging agent doxorubicin **A.** HCT116 p53+/+, null p53, SW480 and HT-29 cells were treated with Dox either singly or combined with SAHA or VPA for 24 hours. Cells were lysed and the proteins separated using SDS-PAGE and detected by WB analysis. The cleavage of PARP, a hallmark of apoptosis and the protein level of HDAC1 and HDAC2 were assessed using the appropriate antibodies. Actin was used as a loading control. **B.** Four cell lines were treated with Dox either singly or combined with SAHA or VPA for 24 hours and cells were lysed and the proteins separated using SDS-PAGE and detected by WB analysis. HDAC2 protein levels were assessed using the appropriate antibodies and quantified using ImageJ software. **C** and **D.** P53 expression vectors were transiently transfected into HT-29 cells and HT29-ShRNA-HDAC2 cells, The cells were treated with Oxa (10μM) for 24 hours. Cells were lysed and the proteins separated using SDS-PAGE and detected by WB analysis. The PARPc and the protein level of p53 were assessed using the appropriate antibodies. Actin was used as a loading control. D) P53 transfected cells were also analyzed for cell death using PI staining and FACS analysis. Cells treated with SAHA+ Oxa or illuminated by UV-C (50 mJ/m2) were used as positive controls. Error bars represent ± S.E.M. of three independent experiments (*n*=3) and statistical significance is depicted by (*) for p<0.05 calculated by a two-tailed Student's T test compared to the control group.

At the 24 hour time point, responses to the applied treatments were classified in terms of sensitivity along the following scale: HCT116 p53+/+ was the most sensitive, then SW480, HCT116 p53−/−, and finally HT-29 (Figure 4A). HDAC2 expression levels increased significantly (P<0.001) with treatment, which correlated with drug resistance (Figure 4A-4B). As HDAC1 and HDAC2 could play compensatory functions in regulating apoptosis, we tested HDAC1 expression level. Our data showed no clear correlation of HDAC1 with cell death in any cell lines (Figure 4A).

To substantiate the importance of HDAC2 in CRC drug resistance, exogenous WT P53 was transfected into HT-29 cells and into HT-29-ShRNA-HDAC2 cells followed by Oxa treatment (Figure 4C). Notably, the introduction of WT P53 did not induce cell death following treatment as examined by the apoptotic end-point of PARP cleavage. Combined treatments were also investigated through the use of Oxa/SAHA in both HT29 and HT-29-ShRNA-HDAC2 cell lines and UV was used as a positive control. Cell death analysis via flow cytometry showed that exogenous WT P53 alone did not sensitise HT-29 to Oxa treatment, rather WT p53 in combination with shRNA-mediated HDAC2 induced treatment sensitivity (Figure 4C and 4D).

**Figure 5:**
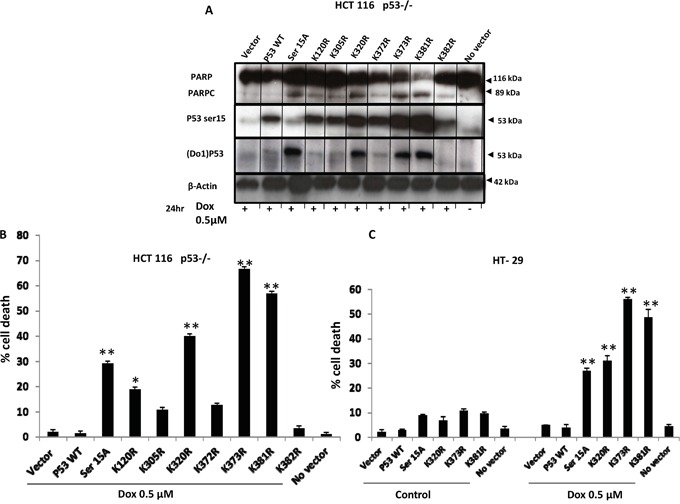
*TP53* mutations (S15A, K120R, K373R and K381R) sensitise HT-29 to Dox treatment **A.** Overexpression of mutations (S15A, K120R, K305R K320R, K372R K373R, and K381Rand K382R) in null P53 HCT-116 cells. All p53 mutants' cells were treated with 0.5μM Dox and cleavage of PARP was detected by WB. **B.** Cell death analysis by flow cytometry. Stable clone HCT116 p53−/− cells were treated with 0.5μM Dox for 24 hours and cell death was assayed by incubating live cells with propidium iodide. **C.** Stable HT-29 cell line was generated from selected mutants (S15A, K120R, K373R and K381R). Cell death analysis was assessed by FACS after 0.5 μM Dox treatment for 24 hours and propidium iodide staining. Error bars represent ± S.E.M.; n=3 independent experiments. Test, t-test, * *for p*<.*005*, ** *for p*<.001.

Functional p53 is crucial for cancer chemosensitivity and associated mutations lead to drug resistance and shorter patient survival in different cancers. Thus, we investigated the significance of p53 mutations in response to the DNA damaging agent Dox. Several stable cell lines with crucial p53 mutations were generated using both null P53 HCT 116 and HT-29 cells. All generated p53 mutants' cells were treated with 0.5μM Dox and cell death was measured by PI staining and flow cytometry analysis. Our data showed that *TP53* WT P53 does not sensitise HT-29 to Dox. Surprisingly, mutations (S15A, K120R, K373R and K381R; Figure 5A-5C) were able to sensitise significantly HT-29 cells to Dox (P<0.001).

**Figure 6:**
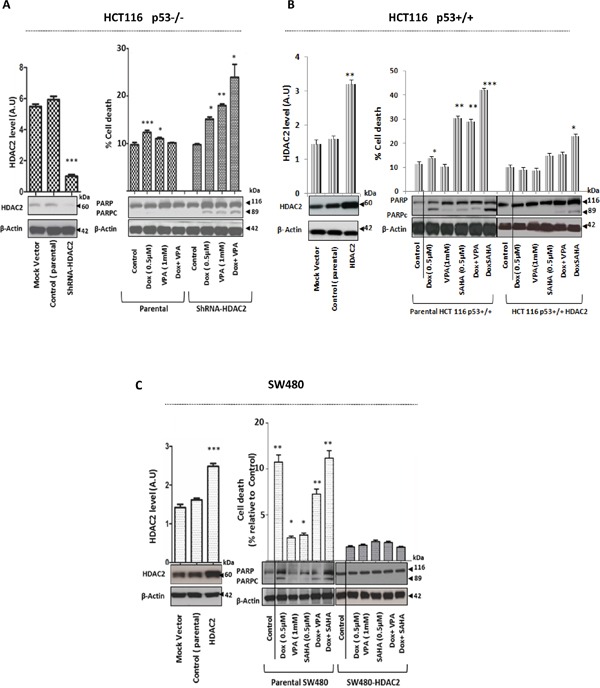
Modulation of HDAC2 expression level by depletion or overexpression directly influences the effect of Dox as single or combined to HDACis in CRC cells **A.** Stable ShRNA-HDAC2 HCT116 p53−/− cells were generated by using lentiviral vector and HDAC2 downregulation was confirmed by WB. Actin was used a loading control. Cells were exposed for 24hr to Dox as single treatment or combined with VPA. Protein cell lysates were separated by SDS-PAGE and detected by WB analysis. The PARPc protein level was assessed using the appropriate antibodies. Actin was used as a loading control. Cells were also harvested and stained by PI to determine cell death using FACS analysis. Error bars represent ± S.E.M. of three independent experiments (*n*=3) and statistical significance is depicted by * for p<0.05 calculated by a two-tailed Student's T test compared to the control group. **B** and **C.** HCT16 p53+/+ and SW480 cell lines were transfected with HDAC2 vector. After 24 hours post-transfection, cells were lysed and the proteins separated using SDS-PAGE. The overexpression of HDAC2 level was detected by WB and quantified using ImageJ densitometry software. HCT16 p53+/+ and SW480 cells transiently transfected with HDAC2 vector were treated with Dox only or combined with VPA or SAHA. After 24 hours, cells were lysed and the proteins separated using SDS-PAGE. The PARPc was analyzed by WB and cell death was quantified by FACS after PI staining. Error bars represent ± S.E.M. of three independent experiments (*n*=3) and statistical significance is depicted by * for p<0.05 calculated by a two-tailed Student's T test compared to the control group.

However, HDAC2 depletion was sufficient to sensitize HCT116 p53−/− cells to Dox and induce cell death (P<0.001) (Figure 6A). Furthermore, transient HDAC2 over-expression in both drug sensitive cell lines HCT 116 p53+/+ (Figure 6B) and mutated *TP53* SW480 cells (Figure 6C) increased the resistance against single and combined treatments significantly (P<0.001).

### HDAC2 controls chromatin plasticity and its depletion enhances mitotic cell death in drug resistant HT-29 cells upon 5-FU and Oxa treatment

**Figure 7:**
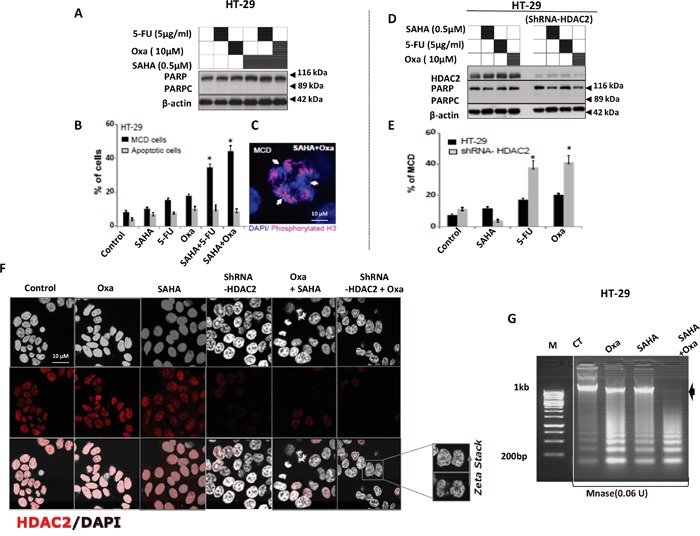
HDAC2 controls the chromatin plasticity and its depletion enhances mitotic cell death in drug resistant HT-29 cells upon 5-FU and Oxa treatments **A.** PARPc measurement in HT-29 treated by 5-FU and Oxa combined with SAHA. After 24 hours, cells were lysed and the proteins separated using SDS-PAGE. **B** and **C.** HT-29 cell lines were treated by 5-FU or Oxa alone or combined with SAHA. After 24 hours, Cells were fixed 4% paraformaldehyde, subsequently DNA was stained with DAPI (0.1 μg/ml; Sigma-Aldrich) and the number of apoptotic cells was measured quantitatively by assessing the percentage of cells with fragmented or condensed nuclei. Mitotic cell death (MCD) was quantified by using phosphorylated histone 3 (ser10) as a mitotic cell marker. C) Representative image of mitotic cell death (MCD) in HT-29 upon SAHA + Oxa combined treatment. **D.** HT-29 or shRNA-HDAC2 HT-29 cells lines were treated with 5-FU or Oxa only or in combination with SAHA. After 24 hours, cells were lysed and the proteins separated using SDS-PAGE. The PARPc and the protein level of HDAC2 were analyzed by WB. Actin was used as a loading control. **E.** HT-29 cell lines or shRNA-HDAC2 HT-29 cells lines were treated by 5-FU, Oxa or SAHA. After 24 hours, mitotic cell death (MCD) was quantified by using phosphorylated histone 3 (ser10) as a mitotic cell marker. **F.** HT-29 cell lines were treated by Oxa alone or combined with SAHA and shRNA-HDAC2 HT-29 cells were treated with Oxa. After 24 hours, cells were fixed and HDAC2 protein was detected after immunofluorescence staining. Nucleus was counterstained using DAPI staining. + z-Stack shows nuclear deformation. **G.** MNase accessibility assay was used to study relaxed chromatin which has higher accessibility to micrococal nuclease enzyme (MNase). Cells HT-29 cells were treated with Oxa or SAHA alone or combined for 24hr and chromatin was extracted and incubated with 0.06U of MNase and fragmented DNA was separated by gel agarose, the arrow represent the undigested DNA. For all the experiments error bars represent ± S.E.M. of three independent experiments (*n*=3) and statistical significance is depicted by * for p<0.05 calculated by a two-tailed Student's T test compared to the control group. Actin was used as a loading control.

We have shown that HT-29 cells were resistant to Dox combined with HDACis. Moreover, WT P53 overexpression was not sufficient to sensitise HT-29 to Oxa, in contrast to exposure UV exposure (positive control), which induced significant PARPc and cell death in both cells (Figure 4C). HT-29 cells showed a significant response to cell death when 5-FU or Oxa was combined with SAHA. Surprisingly, no PARPc was induced (Figure 7A), although there was significant cell death detected by flow cytometry analysis (Figure 2B and Figure 4D). This indicated that cell death induced by both combinations in HT-29 cells did not involve PARPc excluding the apoptotic cell death. Interestingly, we observed that the type of cell death induced in these cells was largely mitotic cell death (MCD) as detected by phosphorylated histone 3 (a mitotic cell marker) (Figure 7A-7C), Interestingly no cell cycle arrest was observed (data not shown).

To investigate the role of HDAC2 in MCD, shRNA-mediated knockdown of HDAC2 was achieved in HT-29 cells. These were then treated with 5-FU, Oxa and SAHA and compared to their parental cells following measurement of both PARPc and MCD (Figure 7D and 7E). HDAC2 knockdown was sufficient to sensitise HT-29 cells to 5-FU or Oxa treatment and only the combined treatments were able to reduce HDAC2 expression as detected by immunohistochemistry similar to HDAC2 knockdown effect (Figure 7F). We noticed that the combined treatment Oxa/SAHA induced chromatin relaxation as assessed by micrococcal nuclease (MNase) assay (Figure 7G). Oxa combined with shRNA-HDAC2 disturbed the higher order chromatin structure in a similar manner to the Oxa combination with SAHA (Figure 7F, Zeta Stack).

### Liposomal-encapsulated Dox/SAHA treatment down-regulates HDAC2 expression in CRC xenografts

**Figure 8:**
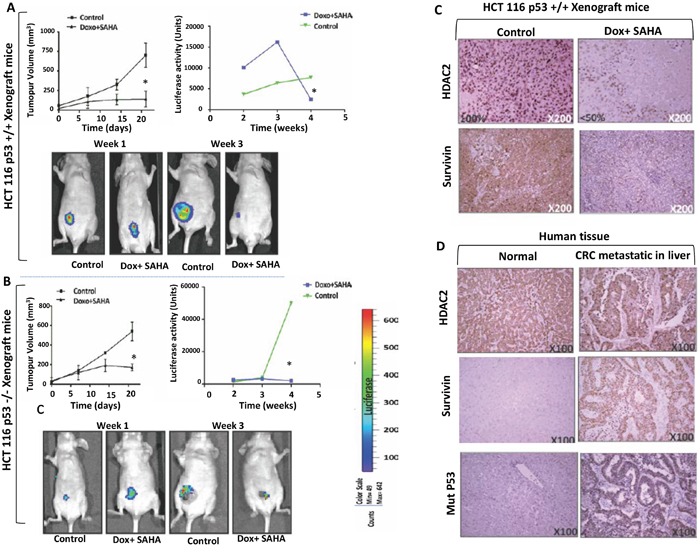
*In vivo* imaging and validation of the effect of combined treatment by liposome-encapsulated SAHA/Dox in xenograft mice **A-B.** HCT116 p53+/+ or HCT116 p53−/− expressing sable luciferase-reporter were transplanted intraperitoneally in male BALB/c nude/nude mice. Liposomal SAHA/Dox-was used to treat HCT116 p53+/+ (n=4) or HCT116 p53−/− (n=4) xenograft mice groups compared to each control groups (n=4). Tumor size manually measured with calipers every three days and bioluminescence imaging measurement every week. Error bars indicate ± SEM (n = 4 replicates). *P <0.005; two-tailed t-test, Imaging was performed by using LICOR. Quantitation of luciferase intensity (error bars indicate ± SEM; n = 4 replicates). P < 0.005, two-tailed t-test). **C.** Protein levels of HDAC2 and survivin in control and treated tumour xenografts of HCT116 P53+/+ were analyzed by immunohistochemistry (HDAC2 expression was found in the nuclei of all control cells and in <50% of treated tumour cells). **D.** Protein levels of HDAC2, survivin, and P53 in normal human liver tissue and liver metastasis of CRC. All antigens were strongly expressed in CRC in contrast to absent or significantly less abundant in normal liver cells. Imaging was performed by using LICOR. (Right) Quantitation of luciferase intensity (error bars indicate ± SEM; *P* < 0.005, two-tailed *t* test, *n* = 4 replicates).

To substantiate the relationship between HDAC2 expression level and the induction of cell death *in vivo* by combined treatment, non-invasive spatiotemporal visualisation was used based on a bioluminescent molecular imaging of a murine xenograft model. Luciferase stable cell lines HCT116 p53 +/+ and HCT116 p53−/− were generated and transplanted into nude mice. Since the combined treatment Dox/SAHA produced significant cell death in both cell lines *in vitro*, the *in vivo* validation of this effect was explored. For this, liposomal-encapsulated Dox/SAHA was prepared and used to treat human CRC xenografts in mice [19]. The size of the tumour was measured before treatment and every three days during the study using callipers. Mice with xenografts of HCT116 p53 +/+ and HCT p53 −/− exhibited a significantly lower tumour volume and luciferase activity in response to the combined treatment (p<0.005) (Figure 8A and 8B). The immunohistochemistry analyzes for HDAC2 expression levels in the tumour isolated from HCT116 p53+/+ xenograft mice showed a more significant decrease after Dox/SAHA combination treatment (P<0.005) (Figure 8C).

**Figure 9:**
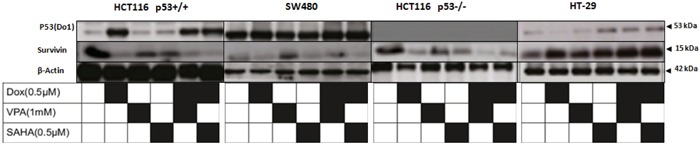
Dox/SAHA combination decreases drastically survivin protein level in HCT 116 p53+/+, HCT116 p53−/− and SW480 but not HT-29 cell lines Four cell lines (HCT 116 p53+/+, HCT116 p53−/− SW480 and HT-29) were treated with Dox, VPA, SAHA or different combinations of these drugs for 24hours. Cells were lysed and the proteins separated using SDS-PAGE and detected by WB analysis. The protein level of Survivin and p53 were assessed using the appropriate antibodies. Actin was used as a loading control.

Survivin, an inhibitor of apoptosis is overexpressed in the majority of cancer types and is associated with chemotherapeutic resistance, reduced apoptosis and increased tumour recurrence. Survivin protein levels were measured by immunohistochemistry (IHC) analyzes of tumours isolated from HCT116 p53+/+ xenograft mice. The results showed a significant decrease in survivin levels after Dox/SAHA combination treatment (P<0.005) (Figure 8C). IHC analyzes of human CRC metastases in liver (n=10) and matched normal human liver tissues (n=10) found higher levels of HDAC2 and survivin in the former compared to the latter (Figure 8D). We also assessed the expression of survivin in HT-29 compared with the other cell lines and showed that following SAHA/Dox or VPA/Dox treatment, there was a significant increase of survivin expression and that this was proportional to a decrease in HDAC2 expression (Figure 9).

### Doxorubicin combined with SAHA or VPA triggers a decrease in histone acetylation

**Figure 10:**
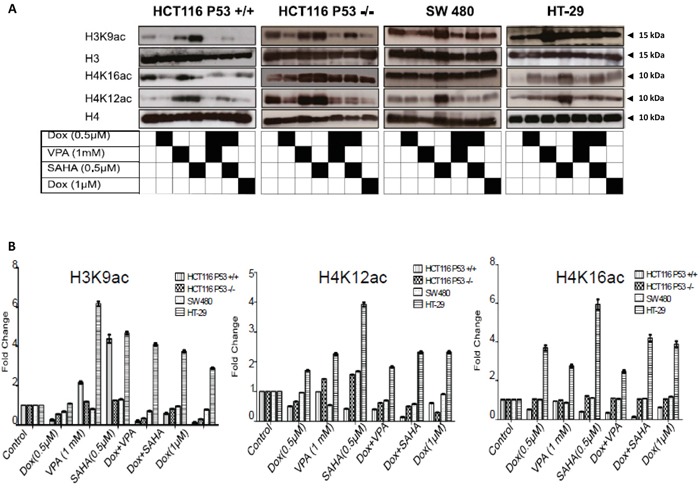
Dox combined with SAHA or VPA triggers induces histone hypo-acetylation **A.** Histones were extracted by acid from the four cell lines (HCT 116 p53+/+, HCT116 p53−/− SW480 and HT-29) after exposure to Dox, VPA, SAHA or different combinations of these drugs for 24hours, also the cells were treated with 1μM. Extracted histones were separated using SDS-PAGE and detected by WB analysis. Histone acetylation levels at residues H3K9 and H4 (K12 and K16) were assessed using the appropriate antibodies. Total H3 and H4 protein levels were used as loading control. **B.** Quantifications of acetylated H3K9, H4K16, H4K12 residues in HCT116 P53+/+, SW480, HCT116 P53+/+, and HT-29 was measured by ImageJ software. Cells were treated only with Dox, VPA or SAHA or by different combinations of these drugs. The changes are presented as fold change in comparison to the control. Error bars represent ± S.E.M.; n=3 independent experiments. One-way ANOVA, Dunnett post-test, P =0.0001.

To determine if there was a correlation between histone acetylation and the sensitivity of CRC cell lines to combined treatment. The levels of histone acetylation in HCT116 p53+/+, HCT116 p53−/− (poorly differentiated) and SW480 (poorly differentiated and invasive) were compared to the well-differentiated CRC cell line HT-29. All cell lines were exposed to Dox, VPA, and SAHA as a single or combined treatment and the changes in histone acetylation established. Dox (1μM) induced significant cell death in HCT116 p53+/+, SW480, HCT116 p53−/− and with no effect on HT-29. Therefore, the effect of combined treatment on histone acetylation was compared using this concentration. Interestingly, Dox combined with SAHA or VPA triggered a decrease in histone acetylation in the poorly differentiated and sensitive CRC cells, but not in the resistant and well-differentiated HT-29 cells. In HT-29 cells, the H3K9ac, H4K12ac and H4K16ac levels remained high after the combined treatments compared to the other cell lines (Figure 10).

## DISCUSSION

We have shown that reduction in HDAC2 expression level plays an essential role in CRC response to DNA damaging agents alone or combined with HDACis. We demonstrated that mutated *TP53* HT-29 cells exhibit extreme resistance to drugs, whilst mutated *TP53* SW480 showed comparable sensitivity to drugs as WT p53 cells. The combined treatment effect was compared with the single treatment alone. All combined treatments tested were found to exert a synergistic effect on cell death in CRC WT p53 cells, whereas cell death induced in null p53 cells shifted from synergistic with Dox/SAHA and CPT/SAHA combinations to antagonistic with Dox/VPA and CPT/NaB combinations. These results point to a limited role for *TP53* in response to Dox/SAHA combination therapy since Dox/SAHA exerted a synergistic effect on cell death in wild type and null p53 cells. In addition, reconstitution of p53 protein in HCT 116 p53−/− and HT-29 cells was not able to increase their sensitivity to drug treatment and, surprisingly, mutated p53 (S15A, K120R, K373R and K381R) sensitised null p53 or HT-29 to Dox.

Reintroducing WTp53 into drug-resistant CRC mutated P53 HT-29 cells does not sensitise cells to conventional CRC treatments Oxa and 5-FU. WTp53 overexpression is considered as a pro-apoptotic protein and several studies showed this is possible when the WTp53 is introduced in functional p53 cancer cells. However, it is not always the case when cancer cells have non functional P53 [20–22], which suggests that the function of mutant P53 could not be rescued when exogenous WTp53 is introduced. Another study demonstrated that the p53 protein expressed in H1299 with no endogenous p53 cells does not seem to function as normal WTp53 despite the fact that DNA damaging agents allow the protein to stabilise and accumulate in the cell [20]. Hence, H1299 cell may have switched to other mechanisms for dealing with DNA damage, since there is no p53 protein expression in H1299 [20]. Therefore, the reconstitution of WTp53 alone is not sufficient to substantially alter the sensitivity of cancer cells to a given chemotherapeutic agent [20–22]. Thus, for the drug regimens used here, *TP53* mutational status may be of less significance in CRC drug resistance.

Previous studies have reported variation in cancer cell death among cells with differing p53 mutational status [22]. For example cells with WT p53, are able to activate AMP-activated protein kinase (AMPK) and protect cancer cells from autophagy to counteract the cytotoxicity caused by the DNA-damaging agent. Conversely, non-functional p53 (mutated) is unable to activate AMPK and cause autophagic cell death [23]. Additionally, PARPc is involved in the molecular mechanisms of various forms of cell death (including apoptosis, necrosis and autophagy) [24]. The appearance of PARPc in the Dox treated mutated cells could cause a similar cell death pathway.

In WT p53, null p53 and mutated p53 (SW480) cell lines treated with SAHA or VPA combined with Dox, a significant decrease in the expression level of HDAC2 is observed when apoptosis is induced, as shown by PARPc or propidium iodide uptake. On the contrary, treatment with the same combinations, MT p53 (HT-29) cell line showed an increase in HDAC2 protein expression with resistance to cell death. Furthermore, HDAC2 overexpression in MT p53 SW480 cells confers resistance to the combined treatment. This suggests that *TP53* mutations in CRC do not confer as much protection against the effect of SAHA or VPA combined with Dox as one would expect, rather it is the HDAC2 protein levels that appear to confer a protective phenotype. Like HDAC2 expression, HDAC1 also seems to be upregulated in CRC [25]. Whilst HDAC2 expression level increased significantly with resistance to treatment, HDAC1 expression had no clear correlation across all cell lines. We also established an *in vivo* model to verify HDAC2 expression levels and to evaluate the response to combined treatments. Liposomal-encapsulated Dox/SAHA was used to treat human CRC xenografts mice as an innovative delivery strategy for toxicity reduction. We found that the combined treatment induced a proportional and significant decrease in HDAC2 expression level, which were associated with tumour shrinkage as measured by tumour size and luciferase activity. Similarly, combined treatment led to a reduction in tumours from both WT p53 and a null p53 cells, again suggesting that p53 is a poor prognostic marker compared to HDAC2 expression.

Deletion of HDAC2 without drug treatment is not sufficient to induce cell death [26]. Here we show that, although HDAC2 depletion is not sufficient for cell death it leads to perturbations in the higher order chromatin structure similar to the effects of HDACis and increased HT-29 sensitivity to 5-FU and Oxa leading to MCD. Whilst the mechanism of HDAC2 inactivation and drug-induced cell death remains largely unknown, recent microarray analysis of HDAC2 inactivation in human hepatocellular carcinoma (HCC) identified a large number of mitotic gene elements including the up-regulation of cell cycle inhibitors and down-regulation of cyclin target genes [27]. The increase of HDAC2 expression has been found in CRC patients at mRNA and protein level indicating that HDAC2 overexpression is due to transcriptional activation [11]. This overexpression of HDAC2 appears to be implicated in cancer through its aberrant recruitment to promoter of tumour suppressor genes leading to gene silencing [28]. HDAC2 transcription is regulated by β-catenin-TCF signalling pathway that is deregulated in CRC. Histone acetyltransferase p300/CREB-binding protein (CBP) is a crucial coactivator for β-catenin-TCF-mediated survivin transcription [28] and the complex regulation of survivin transcription involves enhancement by β-catenin and repression by p53 [29]. As the relationship between survivin and HDAC2 has not been explored in the combinatorial treatment of CRC we investigated this in mice xenograft tumours. Treatment with SAHA/Dox gave a significant reduction in protein expression for both HDAC2 and survivin. Also, we found increased levels of HDAC2 and survivin expression in human CRC metastatic liver tissues.

Compact chromatin is crucial for the protection against agents causing DNA breaks and oxidative DNA damage and this protection is reduced following chromatin relaxation [30]. Using a chromatin relaxation assay, we demonstrated that mutated p53 HT-29 cell line showed relaxed chromatin only after combined Oxa/SAHA treatment. This supports our previous finding, where in the absence of functional p53, SIRT1 (HDAC class III) expression level is a critical parameter in trichostatin A or VPA-mediated sensitisation of several multidrug-resistant cancer cells to the topoisomerase II inhibitor etoposide [2]. HDACis allow for increased histone acetylation and the subsequent chromatin relaxation that occurs may render DNA more susceptible to a number of DNA-damaging agents [31] and increase the binding of transcription factors that regulate genes involved in cell death. Interestingly, it has been suggested that p53 has the biochemical potential for promoting chromatin relaxation, since it can recruit the histone acetyltransferase (HAT) p300 to chromatin. This could facilitate histone acetylation [32] and hence induce chromatin accessibility to enable the efficient detection of lesions [33–35].

Recent studies have shown that the acetylation of H3 or H4 is decreased in poorly differentiated cancer cells while the global expression in moderate to well differentiated cancer cells is increased [36]. However, it is unclear whether such differences in steady state acetylation levels could be indicative of resistance to drugs used singly or combined with HDACis. With this perspective in mind, we demonstrated that Dox combined with SAHA or VPA triggers a decrease in histone acetylation in poorly differentiated and sensitive CRC cells, including SW480 cells, but not in well-differentiated and resistant HT-29 cells. Therefore, in HT-29 the H3K9ac, H4K12ac and H4K16ac levels remain elevated after the combined treatments compared to the other cells. Our results are in agreement with the elevated level of lysine 9-acetylated histone H3 that occurs at the multi-resistance protein 1 (MDR1) promoter in multidrug-resistant cells [37]. Therefore, the association of histone acetylation levels with CRC resistance is of particular importance if the level of acetylation predicts combinatorial therapy outcomes. The level of histone acetylation, however appears to be independent of the deacetylating role of HDAC2 and possibly HDAC2 expression represses transcription factors activation which controls cancer cell death and promotes drug resistance.

It is highly likely that the overall response to conventional treatment may be less robust in patients that strongly express HDAC2 in their cancer cells. Hence, HDAC2 expression in a given tumour might be of prognostic value and also predict the response to combinatorial (HDACis/drug) therapy. Identification of HDAC2 expression as a sensitive “epigenetic biomarker” associated with HDACis/DNA damaging agent's resistance may also lead to a new molecular target for CRC therapy and could help to improve treatment of the disease and provide a more robust mechanistic rationale for the use of HDACis. Further work could present an opportunity to maximise treatment efficacy by pharmaco-epigenetic selection of CRC patients treated with HDAC inhibitors combined with DNA-damaging therapies. Translational protocols for future clinical trials could include HDACis as adjuvant therapeutic agents with conventional CRC drugs.

## MATERIALS AND METHODS

### Cell culture

CRC cell lines: HCT116 P53+/+, HCT116 P53−/−, SW480, and HT-29 were maintained at 37°C in Dulbecco's modified Eagle's medium (Sigma-Aldrich, St. Louis, MO, USA) supplemented with 10% fetal bovine serum (FBS; Sigma) and penicillin/streptomycin in 5% CO2.

### Immunoblotting

Total proteins were extracted from pelleted cells with TGN lysis buffer 50 mMTris-. HCl pH 7.5, 200 mMNaCl, 50 mM sodium β-glycerophosphate, 50 mM Sodium) and subjected to SDS gel electrophoresis. Primary antibodies were used according to the manufacturers' protocols.

### Extraction of acid-soluble nuclear proteins and WB

To detect single acetylated lysine residue on histone H4 and H3 tails, the acid-soluble nuclear proteins were extracted as follow: cells were trypsinased and spun down, washed with phosphate-buffered saline (PBS) and were resuspended in the histone lysis buffer containing 10 mM Tris (pH 6.5), 50 mM sodium bisulfate, 10 mM MgCl2, 8.6% sucrose, 1% Triton X-100 and 0.2 M sulfuric acid completed with protease inhibitors (Sigma). Cells lysates were kept on ice for 1 hour before being cleared by centrifugation at 11,000 g for 30 min at 4°C. The supernatant was precipitated with acetone overnight and then centrifuged at 11,000 g for 1 h at 4°C. Pellets were dried out at room temperature (RT). Acid-extracted histones were obtained by centrifugation at 11 000 g for 1 h at 4°, and the concentrations of the histones were measured by the Bradford method (BioRad) according to the manufacturer's instructions. Histone proteins were resuspended in Laemmli buffer boiled and separated by SDS–polyacrylamide gel electrophoresis (15% gel), Histone proteins were transferred on to nitrocellulose membrane (Amersham, UK). Membranes were blocked for 2 h with 5% non-fat milk in PBS at RT and subsequently probed overnight with primary antibodies raised against histone H3 and H4 or against acetylated histone lysine H3K9, H4K12 and H4K16. The membranes were washed three times in PBS-0.1% Tween and then incubated for 1h with secondary antibodies (HRP-conjugated goat anti-mouse or HRP-conjugated goat anti-rabbit. After three washes 1X PBS with 0.1% Tween 20, bands were detected by enhanced chemiluminescence (ECL Plus) following the manufacturer's instructions (Amersham, UK).

### Reagents and antibodies

Doxorubicin (Dox), 2-propylpentanoic acid (VPA) and sodium butyrate, oxaliplatin (Oxa), 5-fluorouracil (5-FU) were purchased from Sigma-Aldrich (United Kingdom). SAHA (vorinostat) was obtained from Zolinza (Merck, Whitehouse Station, NJ, USA). anti-PUMA was purchased from eBioscience (UK), anti-MDM2 was from Calbiochem, UK, anti-HDAC1, 2, anti-phosphorylated or acetylated P53 and anti-PARP were obtained from Cell Signaling Technology, UK). anti-P53 (Do1) was from GeneTex Inc., USA and anti-Survivin was from Novus Biologicals, USA, and anti-β-actin (Sigma–Aldrich). Anti-histone H3 and anti-H4 (Abcam, Cambridge, UK) or acetylated histone lysine H3K9, H4K12 and H4K16 (Upstate Biotechnology, Billerica, MA, USA). Binding of primary antibodies was detected with a horseradish-peroxidase-conjugated goat anti-mouse (Pierce, Rockford, IL, USA). After repeated washing in phosphate-buffered saline, bands were visualized by enhanced chemiluminescence (ECL Plus) following the manufacturer's instructions (Amersham).

### Preparation of liposomes

Liposomes for *in vivo* therapeutic application were prepared in organic solvent of N1-cholesteryloxycarbonyl-3,7-diazanonane-1,9-diamine (CDAN), 1,2-dioleoyl-sn-glycero-3-phosphocholine (DOPC), cholesterol and 1,2-distearoyl-sn-glycero-3-phosphoethanolamine-N-methoxy(polyethylene glycol)-2000 (DSPE-PEG2000) using a spontaneous vesicle formation procedure (19). The solvent was then removed *in vacuo* to ensure production of an even lipid. The film was re-hydrated and remotely loaded with either 4-(2-hydroxyethyl)-1-piperazineethanesulfonic acid (HEPES) (4 mM, NaCl 135 mM, pH 6.5) or with Doxorubicin and SAHA. The total lipid concentration in the suspension was 2.88 mg/mL. The concentration of doxorubicin and SAHA was selected to approximate a 20g/mouse receiving liposomal doxorubicin at a dosage of 10 mg/kg and a liposomal dosage of SAHA to be 50 mg/kg. The thin- film of nanoparticle liposome was obtained by hydration and by sonication method for 1hour at 30°C, in order to form the required uniform PEGylated liposome, and buffered to a pH of 7.0. The liposomal solution was dialyzed for 18 hours using the Float-A-Lyzer G2 device (Fisher scientific, UK) to remove any un-encapsulated materials (19). The particles had a component molar ratio of 32/24/8/30 (CDAN/DOPC/DSPE-PEG PEG2000/cholesterol). Particle size and zeta potential were determined using a Malvern Zetasizer (Malvern, UK). The size of the vesicles was typically 100 nm.

### Mitotic cell death

The analysis of cells with mitotic catastrophe was based on the quantification of the cells expressing phospho H3 (Ser10) with MTC nuclear morphology in mitosis. Using immunoblotting (antiphospho-Histone H3 (Ser10)) and confocal immunofluorescence analyzes.

### MNase sensitivity assay

Cells were lysed in NP-40 lysis buffer (ce-cold NP-40 lysis buffer (10 mM Tris [pH 7.4], 10 mM NaCl, 3 mM MgCl2, 0.5% NP-40, 0.15 mM spermine, 0.5 mM spermidine) and incubated on ice for 5 minutes.). Nuclei were resuspended in Micrococal nuclease (MNase) digestion buffer(10 mM Tris-HCl pH 8.0, 1 mM CaCl2). A total of 0.06 units of MNase (Sigma-Aldrich, UK) was added to each sample and incubated at 15-20°C for 5 minutes. The reaction was stopped by the addition of MNase digestion buffer, MNase stop buffer ((0.5 ml) - 5% SDS; 250 mM EDTA), proteinase K and 20% SDS followed by overnight incubation. DNA was extracted by standard phenol/chloroform extraction and ethanol precipitation.

### Generation of stable cell lines

Stable cell lines were generated after transfection of p53-null HCT 116 P53−/− or HT-29 cells with wild type P53 plasmid (Origene, UK) or mutant p53 expression plasmids generated by using the QuikChange II site-directed mutagenesis kit (Stratagene) according to the manufacturer. The mutated plasmid were sequenced and verified by using NCB Blast: Nucleotide Sequence. After transfection, the cells that incorporate the plasmids were selected by grown them in medium containing 2.5 μg/ml of puromycin for 2 weeks. The resulting colonies were then pooled and used for further assays than used for experiments.

### Flow cytometry

Cells were trypsinised, washed, and re-suspended in PBS 1Xand distributed in flow cytometry tubes. Cells viability was assessed by staining with Propidium iodide (PI). Cells were then analyzed using FACSCaliburTM(BD Biosciences) machine with the cellQuest program.

### Xenograft *in vivo* models and bioluminescence imaging

Experiments were carried out in compliance with Animals (Scientific Procedure) Act 1986 under the project licence 70/6656. Stable luciferase HCT116 P53+/+ and HCT116 P53−/− clone cell lines were harvested at ~80% confluent, re-suspended in serum-free DMEM and transplanted intraperitoneally in male Balb-c nude/nude mice using a 25 gauge needle. After tumour growth the mice (~14 days after inoculation) were randomly assigned to either the control (n=4) or test conditions (n=4). The size of the tumour in response to the combined treatment was measured before treatment and every three days using callipers. The volume estimated was measured assuming an ellipsoid shape using the following equation:

Volume= length x width x depth x π/6. Animals were sacrificed and the tumours were carefully excised to minimize any surrounding normal tissue. Tumors were weighed, portioned and placed in phosphate-buffered in formalin for (Sigma-Aldrich, UK) for histological evaluation. Luciferase activity in xenografts mice was visualized by luminescence, using the Xenogen IVIS 2000 small-animal *In Vivo* Imaging System (Xenogen Corp., Alameda, CA).

Mice were treated with a dose of 40 mmol/day of Dox/SAHA (200 μL i.p.) combination liposome or 40 mmol/day of HEPES liposome (test) [19] (. Mice were euthanized when severe as cites was observed.

### Human liver samples

Human sample were obtained from the archived material of the Department of Histopathology, Hammersmith Hospital, London, U K, where all the ethics are approved.

### Imunofluoresence

Assays were performed as previously described by us (2). Briefly, Paraformaldehyde- HT-29 fixed cells were incubated with primary antibody (anti-HDAC2 or anti-phosphorylated- H3) and secondary antibody (594 conjugated polyclonal goat anti-mouse IgG antibody). Cell nuclei were counterstained with 4′,6-diamidino-2-phenylindole (DAPI; 1 μg/ml). Samples were mounted with Vectashield (Vector Laboratories, Burlingame, CA, USA) and analyzed with Zeiss 510 META confocal laser scanning microscopy (Zeiss, Jena, Germany).
